# Effectiveness of preemptive antifibrinolysis with tranexamic acid in rheumatoid arthritis patients undergoing total knee arthroplasty: a study protocol for a randomized controlled trial

**DOI:** 10.1186/s12891-020-03488-8

**Published:** 2020-07-15

**Authors:** Yiting Lei, Jiacheng Liu, Xi Liang, Ning Hu, Fuxing Pei, Wei Huang

**Affiliations:** 1 Department of Orthopedics, The First Affiliated Hospital of Chongqing Medical University, Chongqing, 400016 China; 2 Department of Orthopedics, West China Hospital, Sichuan University, Chengdu, 610041 Sichuan China

**Keywords:** Tranexamic acid, Total knee arthroplasty, Rheumatoid arthritis, Preemptive antifibrinolysis

## Abstract

**Background:**

Patients with rheumatoid arthritis (RA) who have undergone total knee arthroplasty are at increased risk of requiring a blood transfusion. This study is designed to compare the effects of preemptive antifibrinolysis of single-dose and repeat-dose tranexamic acid (TXA) in in RA patients undergoing total knee arthroplasty (TKA).

**Methods/design:**

The study will be a double-blind randomized controlled trial with two parallel groups of RA patients. Group A will be given 100 ml normal saline twice daily starting from 3 days before the operation, Group B will be given TXA 1.5 g twice daily starting from 3 days before the operation. All patients will be given TXA 1.5 g 30 min before the operation. The primary outcomes will be evaluated with total blood loss and hidden blood loss. Other outcome measurements such as, fibrinolysis parameters, inflammatory factors, visual analogue scale for post-operative pain, analgesia usage, coagulation parameters, transfusion, the length of stay (LOS), total hospitalization costs, the incidence of thromboembolic events and other complications will be recorded and compared. Recruitment is scheduled to begin on 1 August 2020, and the study will continue until 31 May 2021.

**Discussion:**

In current literature there is a lack of evidence with regard to the efficacy of TXA in RA patients. The findings of this study, whether positive or negative, will contribute to the formulation of further recommendations on the use of TXA in RA patients undergoing TKA.

**Trial registration:**

Chinese Clinical Trial Registry, ChiCTR2000029720. Registered 14 February 2020.

## Background

As the one of the most common inflammatory arthritis affecting about 1% of world population [1, 2], rheumatoid arthritis (RA) is associated with an increasing socioeconomic impact and often affects the diarthrodial joints, including knee joint [3, 4]. Total knee arthroplasty (TKA) is considered one of the most effective orthopedic health care interventions for patients with rheumatoid arthritis (RA) [5–8], and RA patients achieve better satisfaction after TKA compared to patients with osteoarthritis (OA) [8]. However, fibrinolysis stimulated by surgical trauma could lead to significant blood loss [9, 10], and RA patients undergoing this procedure tend to have a higher risk of requiring transfusion than OA patients, owing to preoperative anemia, lower average weight, great bone mass loss, complete synovectomy and extensive soft tissue separation [11, 12]. Blood transfusion is often associated with infectious or noninfectious complications [13], and can prolonged hospital stays, delay recovery and increase medical expenses [14]. Therefore, decreasing blood loss, which can subsequently reduce the requirement for transfusion, is extremely important for patient to improve the quality of life.

As an antifibrinolytic agent, the hemostatic effect of tranexamic acid (TXA) has been proven in TKA [9, 10, 15]. However, in view of the fact that almost 97% of TKAs are performed for OA [16], current published studies in terms of the efficacy of TXA were mainly based on the experience in OA patients [9, 17–20], and there still exists a paucity of evidence with regard to the efficacy of TXA in RA patients. Unlike OA, RA has its own features in the aspects of pathogenesis, prognosis, and medical management [5]. It has been reported that RA patients had higher preoperative D-dimer value than OA patients [21]. Therefore, TXA is theoretically more suitable for RA patients compared to OA patients, and a specially designed TXA scheme for RA patients with TKA is urgently needed.

The most common preoperative protocol of TXA in TKA is to administer one single dose prior to skin incision [9, 22–24], which might seem to be insufficient to inhibit fibrinolysis in RA patients. Therefore, we plan to conduct a randomized clinical trial to compare the effects of preemptive antifibrinolysis of single-dose and repeat-dose TXA in RA patients undergoing TKA.

## Methods

### Study design

**Fig. 1:**
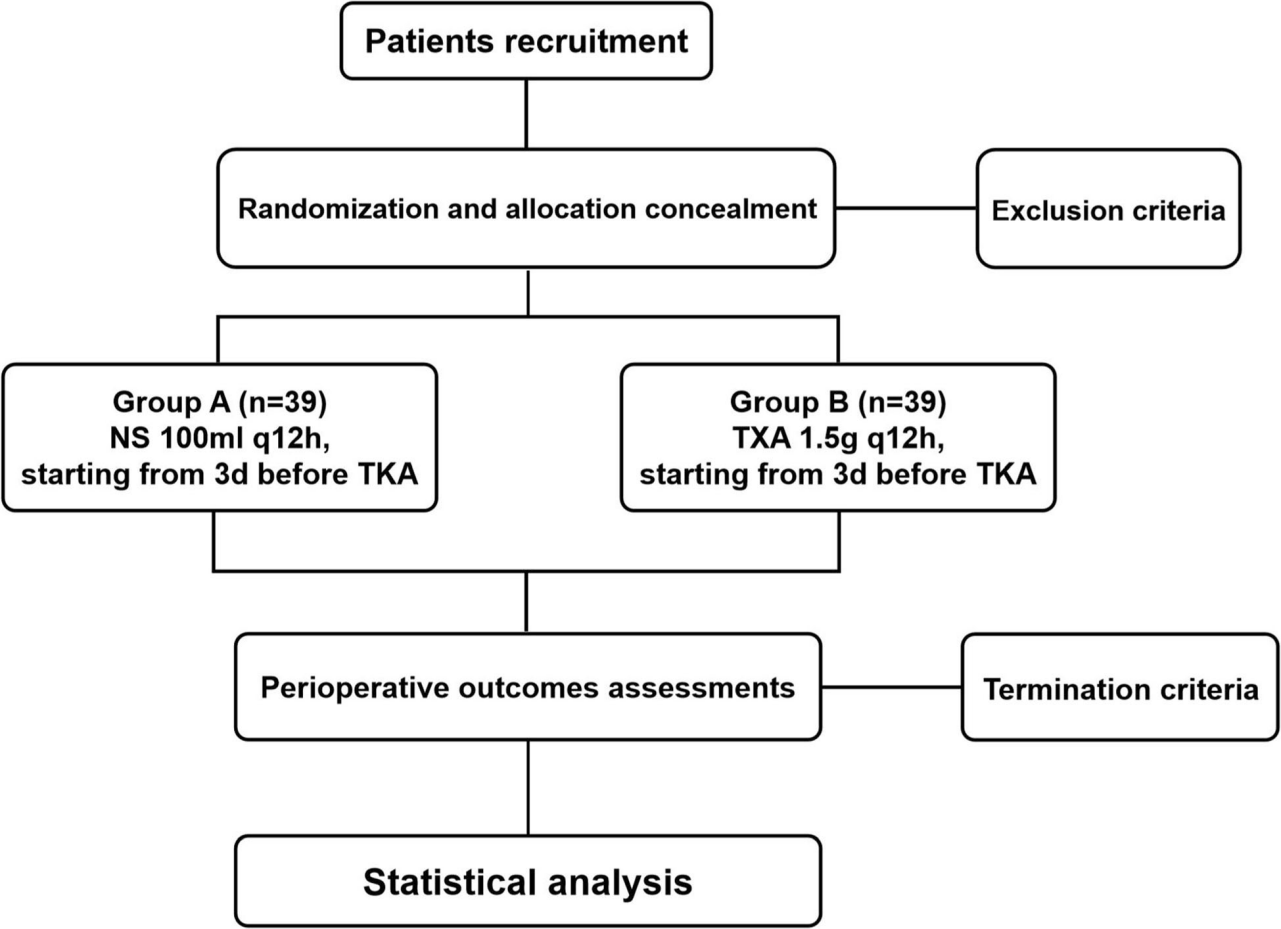
The study flow diagram, including participants recruitment, eligibility, screening, randomization, allocation concealment and outcome assessments. TXA, tranexamic acid

The study will be a randomized, prospective, double-blind (patient and evaluator) clinical trial. This study has been approved, and conforms to the Declaration of Helsinki. The trial has been registered at the Chinese Clinical Trial Registry (ChiCTR2000029798). Informed consent will be obtained. The schedule of trial enrolment and assessments is shown in Table 1. The trial flow chart is shown in Fig. 1.

**Table 1:** The schedule of trial enrolment and assessments

	Study period								
	Pre-D3	Pre-D1	OP	D1	D2	D3	DOD	D14	D90
**Enrolment**									
Eligibility screen	●								
Informed consent	●								
Randomization	●								
**Outcome assessment**									
TBL				●	●	●			
IBL			●						
HBL				●	●	●			
Hemoglobin level	●	●		●	●	●		●	
Fibrinolysis parameters	●	●		●	●	●		●	
Inflammatory markers	●	●		●	●	●		●	
Coagulation parameters	●	●		●	●	●		●	
Pain level	●	●		●	●	●		●	
Analgesia usage				●	●	●			
Knee range of motion	●	●		●	●	●		●	●
LOS						●			
Hospitalization costs						●			
DVT						●		●	●
PE						●		●	●
Adverse events				●	●	●	●	●	●

Pre-D3, 3 days before surgery; Pre-D1, 1 day before surgery; OP, operative; TBL, total blood loss; IBL, intraoperative blood loss; HBL, hidden blood loss; LOS, length of stay; DVP, deep vein thrombosis; PE, pulmonary embolism; D1, the 1st day after surgery; D2, the 2nd day after surgery; D3, the 3rd day after surgery; DOD, the day of discharge; D14, the 14th day after surgery; D90, the 90th day after surgery

### Randomization and blinding

Patients will be randomized into two groups at 1:1 ratio. Randomization will be done based on a computer-generated allocation sequence with random block sizes of 2, 4, and 6, which was stratified by methotrexate use at baseline (0 mg/week, > 0 to < 12.5 mg/week, or ≥ 12.5 mg/week) [25]. Group data will be placed into opaque sealed envelopes. Only nurse and anesthetist who are not involved in this trial will be allowed to check the enrollment and give the corresponding treatment. The outcome evaluators will objectively record the patients’ test results. The surgical team, the study investigators, and the patient will be blinded to interventions until the final data analysis.

### Inclusion criteria

1. The patient is diagnosed with RA in Stage III or IV according to the Kellgren-Lawrence classification [26];
2. The patient will sign informed consent;
3. The patient will undergo the unilateral primary TKA.

### Exclusion criteria

1. A history of a thromboembolic event;
2. Received anticoagulant therapy (warfarin or heparin) within the previous week;
3. Cardiovascular problems or cerebrovascular conditions (history of myocardial infarction, angina, atrial fibrillation, or previous stroke);
4. An acquired or congenital coagulopathy;
5. Allergy to TXA.

### Termination criteria

The patient will be terminated under the following conditions: (1) Shock; (2) Seizure; (3) Digestive disorders, such as nausea, vomiting and diarrhea after administration; (4) Embolic events, such as pulmonary embolism.

### Perioperative anti-rheumatic treatment

Nonbiologic disease-modifying antirheumatic drugs (DMARDs) such as methotrexate, hydroxychloroquine, leflunomide, and/or sulfasalazine, will be used throughout the perioperative period. Biologic agents will be withheld prior to TKA and surgery will be planned at the end of the dosing cycle for that specific medication. The biologic therapy will be restarted when the wound shows evidence of healing without surgical site infections (typically two weeks after TKA) [27].

### Surgery and anesthesia

All the operations will be performed by 2 surgical team composed of 3 senior surgeons (WH, XL and NH) under general anesthesia. The operations will be done in the standard way, with a midline skin incision, a standard medial parapatellar approach, and a measured resection technique. The tourniquet will be applied throughout the whole procedure and will not be released until skin closure. A drainage catheter will be used in every patient for at least 24 h, and will be removed according to the volume of drainage.

### Study interventions

- Group A: 100 mL normal saline (NS) will be administrated intravenously twice daily starting from 3 days before the operation (performed by a nurse). 1.5 g of TXA will be given 30 min before the operation (performed by an anesthetist).
- Group B: 1.5 g of TXA will be given twice daily starting from 3 days before the operation (performed by a nurse). 1.5 g of TXA will be given 30 min before the operation (performed by an anesthetist).

TXA is produced by Chongqing Lummy Pharmaceutical Co., Ltd., and used according to the second edition of 2015 Chinese Pharmacopoeia and Drug Supplement Application Approval YBH19712005; the approval number is National Drug Standard H20056600.

### Antibiotics

1.5g of cefuroxime sodium will be given 30 min preoperatively and every 12 h for 6 times by intravenous drip after the TKA

### Pain management and rehabilitation

The strategies to alleviate pain for all patients will be the same. Intravenous or oral NSAIDs combined with patients-controlled intravenous analgesia (PCIA) will be used after TKA for analgesia. Daily function training (active range of motion training, strength training, and walking training) will be followed out under the supervision and assistance of a physiotherapist.

### Venous thromboembolism prophylaxis

All patients will receive the same routine chemical thromboprophylaxis after operation. Patients will receive 10 mg Rivaroxaban (Xarelto, Bayer, Germany) 12 h postoperatively, repeating at 24-h intervals for 14 days.

### Primary outcomes

#### Perioperative blood loss

The patient’s blood volume (PBV) and perioperative blood loss are calculated according to Nadle et al. [28] and Gross formula [29]:

The patient’s blood volume (PBV) [28] = k1 × height (m) + k2 × weight (kg) + k3 (male: k1 = 0.3669, k2 = 0.03219, k3 = 0.6041; female: k1 = 0.3561, k2 = 0.03308, k3 = 0.1833 for women).

The total blood loss (TBL) [29] = PBV × (Hct_pre_ − Hct_post_)/ Hct_ave_ (Hct_pre_ = preoperative hematocrit level. Hct_post_ = the lowest postoperative hematocrit level during hospitalization. Hct_ave_ = the average of the Hct_pre_ and Hct_post_).

The intraoperative blood loss (IBL) [30] = (the weight of the gauze pads and compresses with blood – the dry weight of the gauze pads and compresses) + (the volume collected by the aspirators – the volume used to wash the surgical area).

The hidden blood loss (HBL) [31] = TBL – measured blood loss (measured blood loss mainly included IBL and the volume of drainage).

### Secondary outcomes

#### Fibrinolysis parameters, inflammatory markers and coagulation parameters

Fibrinolysis parameters [fibrin(−ogen) degradation products (FDP), D-dimer], inflammatory markers [erythrocyte sedimentation rate (ESR), C-reactive protein (CRP), interleukin-6 (IL-6)] and coagulation parameters [activated partial thromboplastin time (APTT), prothrombin time (PT) and thrombelastography (TEG)] will be tested 1 and 3 days before surgery and 1, 2, 3 and 14 days after surgery. All measurements will be performed by the Department of Clinical Laboratory of The First Affiliated Hospital of Chongqing Medical University.

#### Post-operative pain score and analgesia usage

Pain level at rest (rest in bed at least for 30 min before test) and during activity (from sitting to standing position) will be assessed using a visual analogue scale (0 means no pain, 10 means severe pain imaginable) and will be conducted 1 and 3 days before surgery and 1, 2, 3 and 14 days after surgery, and the analgesia usage will be carefully recorded.

#### Transfusion and knee function

Transfusion will be carefully recorded during hospitalization and follow-up period. The criterion of blood transfusion in our medical institution will be in accordance with the guidelines of the National Ministry of Health [9]: (1) the hemoglobin (Hb) level is equal to or less than 70 g/L; (2) the Hb level is between 70 g/L and 100 g/L, with intolerable symptom of anemia (dizziness, tachycardia, tachypnea, or decreased exercise tolerance).

The knee range of motion will be measured preoperatively and 1, 2, 3, 14 and 90 days after TKA, and the knee angle will be measured by goniometer.

#### Length of stay and total hospitalization costs

The LOS will be recorded from the day of admission to discharge. Total hospitalization cost will be defined as the total payment that the patients’ primary insurance carrier provides to the hospital.

#### Adverse events

Each patient will be screened with clinical symptoms (acute onset, swelling, sever pain) and physical examination (significant tenderness at the femoral triangle or/and leg) to ensure no deep vein thrombosis (DVT) is present. Doppler ultrasound in diagnosis of venous thrombosis of lower extremities will be performed at the time of hospital discharge, and repeated at 2 weeks and 3 months after TKA.

Pulmonary embolism (PE) will be investigated by clinical signs and symptoms (cough, hemoptysis, chest tightness, tachypnea, shock, dizziness, cyanosis, etc.). Patient with clinically suspected PE will undergo a contrast-enhanced chest CT scan immediately.

The wound complications (ecchymosis, bleeding, local hematoma, superficial and deep infection) will be carefully recorded during hospitalization and follow-up period.

### Data management

Each participant’s identification number and baseline data will be electronically contained in CRFs. Outcome data will be put in the CRF by two independent trained research assistants with a double-entry method. No personal data will be shared with any other outside party.

### Sample size calculation

Sample size was estimated based on a large database generated from a prospective multicenter study sponsored by the Chinese Health Ministry (project number 201302007) using PASS 2011 (NCSS, LLC. Kaysville, Utah, USA) software. According to the database, the TBL was 730.96 ± 262.85 mL, with a single dose of TXA before TKA in RA patients. To detect a difference of 200 mL of primary end point, with a power of 0.90 and the significance level of 0.05, 31 patients per arm will be needed. Considering a dropout rate of 20%, we decided to involve 39 patients in each arm.

### Statistical analysis

Statistical analyses will be performed using SPSS version 24 (SPSS Inc. USA) software. We will use the independent samples t-test (Student’s t-test) to evaluate quantitative data with normal distribution, and use Mann–Whitney U-test for data not having normal distribution. Pearson Chi-square test or Fisher exact test will be used to analyze qualitative comparative data. *P* values will be judged significant if they are less than 0.05.

### Ethics and dissemination

This clinical trial has been approved by the ethics committee of the First Affiliated Hospital of Chongqing Medical University (2019–015). The data will be published on the website of the China Clinical Trials Registry and the results of this study will be published in a peer-reviewed journal in accordance with CONSORT, and disseminated at relevant national and international conferences.

## Discussion

To our best knowledge, this will be one of the first attempt to compare the effects of preemptive antifibrinolysis of single-dose and repeat-dose TXA in RA patients undergoing TKA. The hemostatic effect of TXA has been well-established in OA patients undergoing TKA [9, 10, 32]. However, the efficacy and safety of TXA in RA patients is still undetermined. Compared with OA patients, RA patients are at a higher risk of requiring blood transfusion [11, 12]. Mukubo et al. found that D-dimer in RA patients was ten times normal, indicating a hyperfibrinolytic state in RA patients prior to surgery [21]. In addition, fibrinolysis can also be caused by surgical trauma and enhanced by tourniquet [9]. Since fibrinolytic activation is a cascade process and is most easily inhibited at its early stage [33], the preoperative dose has been deemed as the most significant dose [23, 34]. Hourlier et al. indicated that the reason why the previous studies failed to show the efficacy of single-dose treatment may owe to the lack of dosage [35]. Therefore, we assume that repeat-dose preoperative TXA may exert an auxiliary antifibrinolytic effect. In this trial, different preoperative dosing regimen will be compared, which will contribute to the formulation of further recommendations on the use of TXA for reducing blood loss in RA patients undergoing TKA.

RA is characterized by persistent synovitis and systemic inflammation, which may lead to joint deformity and disability [36, 37]. In addition, since inflammation and atherosclerosis are closely linked, it is reported that satisfactory inflammation control is likely to decrease the cardiovascular risk in patients with RA [38]. Despite numerous studies proving the effect of TXA on fibrinolysis, it still remains unclear in terms of its anti-inflammatory properties [9, 17, 39]. Data from previous studies showed that D-dimer could stimulate monocytes to synthesize and release IL-6, indicating that there is a close connection between fibrinolysis and inflammation [40, 41]. Jimenez et al. found that double-dose TXA could prolong the inhibition of fibrinolysis and alleviate postoperative inflammation in cardiopulmonary bypass (CPB) patients [42]. However, evidence is scarce in RA patients with regard to the anti-inflammatory effect of TXA. In this trial, we will compare different preoperative TXA regimens, suspecting that repeat-dose preoperative TXA could provide additional inflammation control.

A number of previous studies have demonstrated the presence of preoperative blood hypercoagulability in RA patients, which is a risk factor of postoperative venous thromboembolism (VTE) [43]. In addition, concerns about the potential pro-coagulation effect of TXA in this high-risk patient population still hinder the wide application of this medication in TKA [44]. Therefore, we plan a prospective randomized controlled study to evaluate the safety of different TXA regimens in RA patients undergoing TKA. In this study, we will use APTT, PT and TEG to detect perioperative blood hypercoagulability in patients with RA, which may provide some insights to assess the safety of TXA in RA patients.

## Data Availability

Not applicable.

## Abbreviations

RA: Rheumatoid Arthritis
TXA: Tranexamic Acid
TKA: Total Knee Arthroplasty
LOS: Length of Stay
OA: Osteoarthritis
DMARDs: Nonbiologic Disease-modifying Antirheumatic Drugs
NS: Normal Saline
PCIA: Patients-controlled Intravenous Analgesia
PBV: Patient’s Blood Volume
TBL: Total Blood Loss
IBL: Intraoperative Blood Loss
HBL: Hidden Blood Loss
FDP: Fibrin(−ogen) Degradation Products
ESR: Erythrocyte Sedimentation Rate
CRP: C-reactive Protein
IL-6: Interleukin-6
APTT: Activated Partial Thromboplastin Time
PT: Prothrombin Time
TEG: Thrombelastography
Hb: Hemoglobin
DVT: Deep Vein Thrombosis
PE: Pulmonary Embolism
CONSORT: Consolidated Standards of Reporting Trials
